# Biocompatibility and Effectiveness Evaluation of a New Hemostatic Embolization Agent: Thrombin Loaded Alginate Calcium Microsphere

**DOI:** 10.1155/2017/1875258

**Published:** 2017-02-16

**Authors:** Fengqi Xuan, Jingjing Rong, Ming Liang, Xuwen Zhang, Jingyang Sun, Lijun Zhao, Yang Li, Dan Liu, Fei Li, Xiaozeng Wang, Yaling Han

**Affiliations:** ^1^Department of Cardiology, The General Hospital of Shenyang Military Region, Shenyang, Liaoning 110016, China; ^2^Electrocardiogram Laboratory, Dezhou People's Hospital, Dezhou, Shandong 253000, China; ^3^Department of Ophthalmology, Dezhou People's Hospital, Dezhou, Shandong 253000, China

## Abstract

*Background*. Until now, there has been no ideal embolization agent for hemorrhage in interventional treatment. In this study, the thrombin was encapsulated in alginate calcium microsphere using electrostatic droplet technique to produce new embolization agent: thrombin loaded alginate calcium microspheres (TACMs).* Objectives*. The present work was to evaluate the biocompatibility and hemostatic efficiency of TACMs.* Methods. *Cell cytotoxicity, hemolysis, and superselective embolization of dog liver arteries were performed to investigate the biocompatibility of TACMs. To clarify the embolic effect of TACMs mixed thrombus in vivo, hepatic artery injury animal model of 6 beagles was established and transcatheter artery embolization for bleeding was performed.* Results*. Coculture with VECs revealed the noncytotoxicity of TACMs, and the hemolysis experiment was negligible. Moreover, the histological study of TACMs in liver blood vessel showed signs of a slight inflammatory reaction. The results of transcatheter application of TACMs mixed thrombus for bleeding showed that the blood flow was shut down completely after the TACMs mixed thrombus was delivered and the postprocedural survival rate of animal models at 12 weeks was 100%.* Conclusions*. With their good biocompatibility and superior hemostatic efficiency, TACMs might be a promising new hemostatic agent with a wide range of potential applications.

## 1. Introduction

With the rapid development of vascular intervention, hemostatic management of blunt abdominal trauma (such as spleen and liver) has been transformed from traditional surgery to transcatheter arterial embolization (TAE) [1–3]. One of the most challenging decisions in TAE is to choose the appropriate embolic agent. To date, the commonly used embolic materials for hemostasis in abdominal trauma include coils, GELFOAM, and liquids agent [4, 5]. However, coils may result in potential complications such as nontarget embolization, migration, or damage to vessel [4]. GELFOAM, a kind of nonspherical particles, is unable to precisely control the location of embolization. Besides, the recanalization time of GELFOAM is unpredictable as a temporary embolic agent [6, 7]. In addition, Onyx is an expensive liquid embolic agent and may cause severe vasospasm if injected rapidly [8, 9]. Until now, there are no ideal embolic materials for interventional hemostasis of blunt trauma and hemorrhage of solid abdominal viscera.

Alginate, which was obtained from brown algae, was widely used as natural polymers because of its good biocompatibility, biodegradation potential, nontoxicity, and easy availability [10–12]. Alginate calcium microspheres have been particularly attractive in the biomedical research field and have been subsequently employed as an embolic agent for treating aneurysms, tumors, or uterine fibroids successfully. These microspheres were considered as embolic agents due to their favorable properties, including drug delivery function, mechanical stability, and nonadhesive characteristics [13–15]. As a hemostatic drug, thrombin could produce a fast hemostatic effect by inducing acceleration of platelets and authorizing the conversion of fibrinogen into fibrin, but autologous clot has the inherent weakness of rapid lysis and restoring circulation within hours. In this study, we put forward an idea for preparing a kind of new embolic material, thrombin loaded alginate calcium microspheres (TACMs), which combine the advantage of embolic microspheres with the efficient procoagulant activity of thrombin. Rong et al. have previously developed a novel application method of TACMs by mixing TACMs with whole blood in vitro to form a stronger mixed thrombus as embolic agent [16]. This study was conducted to evaluate biocompatibility of TACMs both in vitro and in vivo. And then the hemostasis effect of TACMs in solid organ bleeding was validated in the long run.

In this research, TACMs were prepared using electrostatic droplet techniques, and morphology, size distribution, cytotoxicity test, and hemolysis assays of TACMs were performed in vitro. To further explore the biocompatibility of TACMs in vivo, a model of canine hepatic artery embolization was developed, and the toxic effects as well as tissue responses were evaluated. In addition, we established hepatic artery injury animal model of 6 beagles to clarify the embolic effect of TACMs mixed thrombus.

## 2. Materials and Methods

### 2.1. Materials

Sodium alginate (purity ≥ 98%, viscosity = 100 cp, average molecular weight = 400 kDa, and G/M = 0.38) was purchased from Bright Moon Seaweed Group Co., Ltd. (Qingdao, China). Thrombin derived from swine or bovine blood was purchased from First Biochemical Pharmaceutical Co., Ltd. (purity ≥ 80%, Shanghai, China). Human arterial vascular endothelial cells (HAVECs) were purchased from American Tissue Culture Collection (ATCC), Cell Counting Kit-8 (CCK-8) was purchased from Beyotime (Shanghai, China), calcein-AM from DOJINDO (Japan), and Propidium Iodide (PI) from Sigma (Germany).

Animal experiments in our research were performed in accordance with the regulations of the ethical committee of our hospital for animal experiments, and all animals received humane care in accordance with the guideline published by the National Society for Medical Research (Principles of Laboratory Animal Care) and by the National Institutes of Health (Guide for the Care and Use of Laboratory Animal, NIH publication number 85-23, revised 1985).

### 2.2. Preparation of Microspheres

The microspheres were prepared using electrostatic droplet technique, following the following steps: 2.5% (W/V) alginate solution was made by dissolving sodium alginate in 0.9% (W/V) normal saline, and then the solution was filtered through 0.8, 0.45, and 0.22 *μ*m membrane filters to remove bacteria. The thrombin was then added into the alginate sodium solution at an ultimate concentration of 12 mg/mL. The mixed solution was injected through a needle into a gel solution of 2% (W/V) CaCl_2_ using an electrostatic droplet generator (YD-06, Dalian Institute of Chemical Physics, Chinese Academy of Sciences, China), forming alginate calcium microspheres containing thrombin. And the parameters of device were set as follows: voltage, 6.5 kV, frequency, 160.3 Hz, syringe needle specification, 4.5#, and pump speed, 5.8 mL/hour.

### 2.3. Morphological Examination and Size Distribution of TACMs

The internal and surface structures of microspheres were observed using scanning electron microscopy (SEM, JEOL JCM-5000, Japan). Freshly prepared TACMs were fixed by 4% paraformaldehyde and then subjected to dehydration for 15 min each in an increasing series of ethanol (50%, 70%, 90%, and 100%). Subsequently, the sample was dried in vacuum using freeze-drying method. The SEM was used after the samples were coated with a thin gold layer. Size distribution of TACMs was determined with a light microscope (Leica DM3000, Germany). The diameter of randomly selected 500 microspheres was measured, and the size distribution was described with frequency histogram.

### 2.4. Cytotoxicity Test

#### 2.4.1. Cytotoxicity of Microsphere Extracts

2 g microspheres were mixed with 10 mL complete medium (DMEM medium containing 10% new born calf serum, 50 ug/mL streptomycin, and 50 units/mL penicillin) at 37°C for 24 h in 5% CO_2_ atmosphere. The supernatant was filtered through a hydrophilic nylon net Millipore membrane (pore size: 20.0 *μ*m) and three different extract concentrations (100%, 50%, and 25%) were prepared [17]. The sample was prepared according to ISO 10993-12 standard [18]. HAVECs were maintained in complete medium at 37°C in a humidified incubator and seeded in a 96-well plate at a density of 5000 cells per well. When cells were attached completely, the culture medium was removed and the microsphere extract (100%, 50%, and 25%) was added immediately. Cell viability was quantified using CCK-8 assay. At predetermined time points (24, 48, and 72 h), 10 *μ*L of CCK-8 was added to each well and the samples were incubated for 2 h at 37°C before their optical density was measured at 450 nm using a multimode microplate reader (Bio-Rad 550, USA). The relative growth rate (RGR) was calculated using the following equation:

(1) RGR  %=OD  experimental  group−OD  blank  groupOD  control  group−OD  blank  group×100%,

where OD (experimental group), OD (blank group), and OD (control group) are the absorbance of the experimental group, blank group (media without any cells), and the control group (media incubated with normal cells), respectively.

#### 2.4.2. Contact Toxicity

Microspheres (60–80 per well) were cocultured with HAVECs (2 × 10^4^ per well) in a 24-well plate under the same conditions as mentioned in Section 2.4.1. At time points of 24 h and 48 h, the microspheres were removed from the wells and the wells were washed twice with PBS. Then the cells were stained with the live/dead viability assay using calcein-AM and PI [19, 20]. Using the inverted fluorescence microscope (Olympus IX-70, Japan), live cells were stained green by calcein-AM and dead cells were stained red by PI under 490 nm excitation wavelength. Five images were taken randomly per well by the same operator and the numbers of live and dead cells were recorded separately. Cell survival rate above 80% was considered to be acceptable.

### 2.5. Hemolysis Assay In Vitro

The hemolytic potentials of TACMs were measured by a universal method similar to the earlier report [21, 22]. 30 healthy volunteers were recruited with approval from Medicine Research Ethics Committee of the General Hospital of Shenyang Military Region. All subjects provided written informed consent, and the study was carried out in accordance with institutional guidelines. Healthy human blood containing sodium citrate (3.8 wt.%) with the volume ratio of 9 : 1 was centrifuged at 1000 r for 5 min and washed using normal saline three times to produce 2% RBC solution. TACMs (1.25 mg, 2.5 mg, 5 mg, and 10 mg) were added to the RBC solution (2 mL) and the mixtures were incubated at 37°C in a water bath for 2 h. Positive and negative controls were produced by adding RBC to distilled water and saline water, respectively. After incubation, all samples were centrifuged and the supernatant was measured at 545 nm using spectroscopic analysis. The percentage of hemolysis was calculated as follows [23]:

(2) hemolysis%=OD  test−OD  negative  controlOD  positive  control−OD  negative  control×100%.

### 2.6. Biocompatibility of TACMs In Vivo

#### 2.6.1. Liver Artery Embolization with TACMs

Fifteen animals (beagle, male, 20–25 kg) were divided into three follow-up groups: 72 h, 4 w, and 12 w groups. Before the procedure, anesthesia of animals was induced with intravenous propofol (3 mg/kg) and maintained with a continuous intravenous infusion of propofol (10 mg/kg/h). The animals were placed in the supine position with the hind legs extended. Heart rate and rhythm were monitored by a continuous 3-lead electrocardiogram. After puncture of the femoral artery with the puncture needle, a 6F short sheath was introduced. Then an angiography catheter (6F, RH*∗*5TIG110M, Terumo, Tokyo, Japan) was used to obtain an arteriogram of the hepatic vasculature. Selected catheterization of the right liver artery was performed using a microcatheter (FINECROSS, NC-F865A, Terumo, Tokyo, Japan). Then the suspension of TACMs and contrast agent (Iopromide, Bayer, China) with the volume ratio of 1 : 3 was injected from 10.0 mL syringe into the microcatheter. The procedure was not ended until the stasis of flow lasted at least 5 s. The whole embolization progress was controlled fluoroscopically.

#### 2.6.2. Toxicity Evaluation

At the beginning of operation and at 1 d, 3 d, 5 d, 7 d, 20 d, 30 d, 60 d, and 90 d after operation, the blood samples were taken using the vein puncture to analyze the alanine aminotransferase (ALT) and aspartate aminotransferase (AST), which were considered as indicators of hepatocellular toxicity. Meanwhile, red blood cell (RBC), haemoglobin (HB), and hematocrit (HCT) were considered as indicators of hemolysis and white blood cell (WBC) was considered as an indicator of inflammation. After blood sampling, the weight and body temperature of animals were measured.

The animals in different groups were sacrificed with a lethal dose of propofol at the predetermined time points (72 h, 4 w, and 12 w after operation). The embolized liver was surgically removed and fixed in 4% formaldehyde. Tissue samples from the liver near the embolization site were taken in order to evaluate the tissue reaction. Each sample was embedded in paraffin and 4 *μ*m thick sections were prepared. Staining with haematoxylin-eosin (H&E) was histologically observed under a microscope by an experienced pathobiologist. Banff 97 scoring system was applied to classify the inflammatory changes and giant cell score was used to quantify the extent of foreign body reactions after TACMs embolization [24]. The detailed evaluation criteria of Banff 97 score and giant cell score were shown in Table 1.

### 2.7. The Embolic Effect of TACMs Mixed Thrombus In Vivo

To clarify the embolic effect of TACMs mixed thrombus in vivo, hepatic artery injury animal model of beagles was established. Six beagles (male, 20.0–25.0 kg) were used in this study.

**Figure 1:**
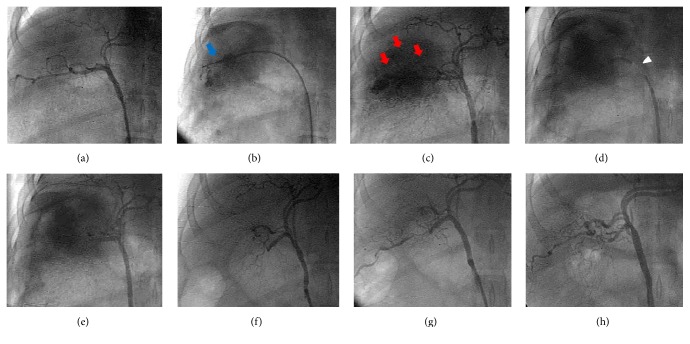
The establishment of hepatic injured and hemorrhagic models of beagles and the interventional embolization using TACMs mixed thrombus. (a) The angiography of the normal hepatic artery; (b) the hemorrhage model made by a Swartz sheath (blue arrow); (c) the continuous leakage of contrast agent from injured right hepatic artery 5 minutes after hemorrhage model was established (red arrow); (d) the delivery of TACMs mixed thrombus (white arrow) by guiding catheter; (e) bleeding stopped completely after embolization; (f) angiography 1 week later after embolization; (g) angiography 4 weeks later after embolization; (h) angiography 12 weeks later after embolization.

After anesthesia was induced by intraperitoneal injection of chloral hydrate (3 mL/kg) in catheter lab, endotracheal intubation was performed and the anesthesia was maintained at 10 mg/kg/h and the vital signs of animal were continually monitored during the operation. The right common femoral artery of animal was surgically exposed and a 6F sheath was placed in it. 100 U/kg heparin was injected in the body from the sheath. Then, 3 mL whole blood was drawn from the artery sheath and was rapidly mixed with 0.5 mL TACMs in a 5 mL syringe to form TACMs and whole blood mixed clot as embolic agent. In order to site-specifically release the embolic agent with no complications related to the procedure, a special delivery method named “sandwich delivery” was performed [16]. By using guide wire, an angiography catheter was inserted along the sheath into the hepatic artery to perform angiography (Figure 1(a)). Then, a Swartz sheath was inserted along the catheter, with the tip positioned against the predetermined site in the right hepatic artery wall. And then the Swartz sheath was pushed forward repeatedly to damage the artery for establishing the injury model (Figure 1(b)). Angiography was performed 5 minutes later to confirm the injury based on the serious continued leakage of contrast agent from the right hepatic artery (Figure 1(c)). Then, a guiding catheter was located into the embolism site (the proximal portion of injured right hepatic artery) and the TACMs mixed thrombus (embolic agent) was released (Figure 1(d)). The dose of embolic agent was determined by the stopping of bleeding from the right hepatic artery, which was monitored closely by angiography (Figure 1(e)). Animals were then sent back to the experimental animal center for observation after operations, and follow-up angiography was performed at 1, 4, and 12 weeks to observe the morphological changes of injured hepatic artery and liver (Figures 1(f)–1(h)).

### 2.8. Statistical Analysis

The data were summarized using the mean and standard deviation for numeric variables for every single specimen and study group. For data comparison, the nonparametric Kruskal-Wallis *H* test was applied. Difference was considered significant if *p* value was less than 0.05. All statistical analyses were performed using the Statistical Package for Social Sciences 21.0.

## 3. Results and Discussion

### 3.1. Morphological Examination and Size Distribution of TACMs

**Figure 2:**
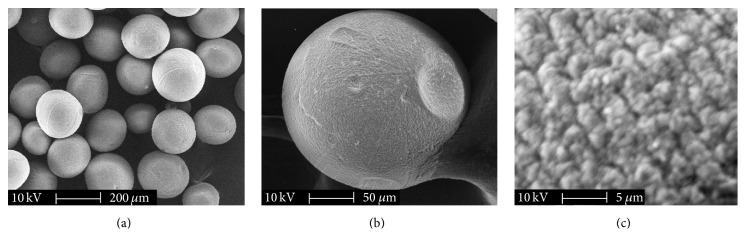
SEM micrograph of the TACMs. (a) A cluster of microspheres; (b) a whole microsphere; (c) surface of the microsphere.

**Figure 3:**
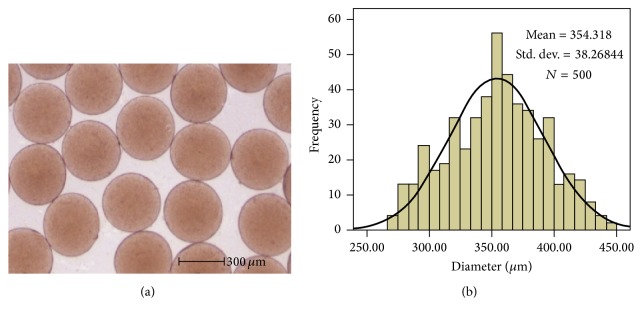
Size distribution of TACMs. (a) Optical microscope images of TACMs; (b) corresponding size distribution of the microspheres.

The TACMs were successfully prepared under very mild conditions without high temperatures or covalent cross-linking agents using electrostatic droplet technique.  Figure 2 shows images of TACMs under different magnifications by SEM. It demonstrated that TACM was a typical generic sphere in shape and the surface was rough and porous. These characteristics were in favor of diffusion of thrombin out of microspheres.  Figure 3(a) shows representative optical microscope photographs of TACMs and Figure 3(b) describes size distribution. Microspheres appeared as spherical compressible entities and displayed a relatively uniform size. In this study, the TACMs with an average size of 350 *μ*m were investigated.

### 3.2. Cytotoxicity Test

**Figure 4:**
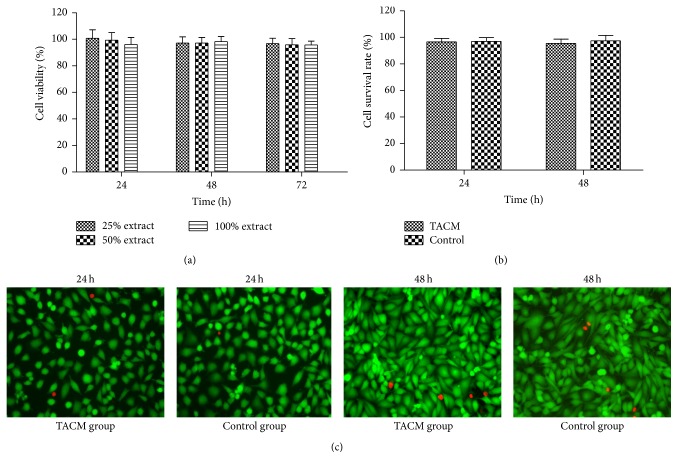
Cytotoxicity assay of TACMs. (a) Cell viability of HAVECs grown with different concentrations of microsphere extracts and merely culture medium at 24, 48, and 72 h after incubation. Values were represented as mean ± standard error of the mean (*n* = 8). VECs viability was maintained over 90% at predetermined time points. (b) Cell survival rates of VECs contacting with TACMs for 24 h and 48 h (*n* = 8); the control group was VECs with culture medium alone. There was no significant difference between TACM group and control group (*p* > 0.05). (c) VECs were stained with calcein-AM and PI at 24 h and 48 h, and cells with green and red fluorescence represented the live and dead cells, respectively.

Cytotoxicity analysis in vitro plays a vital role in biocompatibility evaluation because of its high sensibility, convenience, and not being influenced by internal environment [26, 27]. TACMs was used as a kind of vessel embolization material in this research; thus we chose HAVEC lines as experimental cells due to their similarities to the endothelial layer of human vessel [28]. As depicted in Figure 4(a), the results from CCK-8 assay showed that more than 90% of HAVECs were metabolically active after contacting with different concentrations of extracts after 24 h, 48 h, and 72 h of seeding. According to the standard of toxicity rating, the cell toxicity of the TACMs was in grade I or grade 0 (grade I: RGR within 75–99%; grade 0: RGR ≥ 100%), which is considered a low toxicity and is in the range of safety use [29, 30]. In live/dead fluorescence viability testing, TACMs contacted with VECs directly, which was out of consideration of the physical interaction of TACMs with VECs. Figures 4(b) and 4(c) illustrate that the average cell survival rates in TACM and control groups were 96.7% and 97.0% at 24 h and 95.6% and 97.4% at 48 h, respectively, and no significant difference was found between them throughout the observation period (*p* > 0.05). Based on the results above, we concluded that the TACMs and their ultimate degradation byproducts were noncytotoxic and may not induce membrane damage or impairment of metabolic activity to HAVECs.

### 3.3. Hemolysis Test

**Figure 5:**
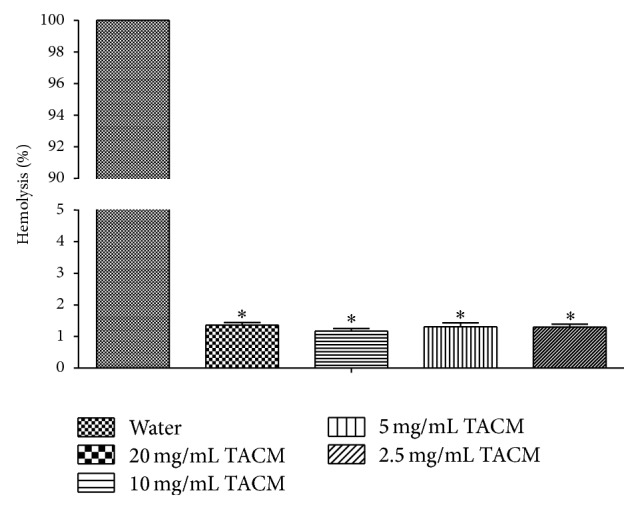
Hemolysis test of RBC following incubation with TACMs. Hemolysis percentages of TACMs samples were all less than 5%, and the positive controls were 100%. ^*∗*^Corresponds to a *p* < 0.05 relative to positive control.

Blood compatibility is a very important biological characteristic for a new kind of embolic material on account of direct contacting with blood. Hemolysis test is considered as a simple and reliable measure to estimate hemocompatibility [30] and the hemolysis rate lower than 5% is permissible [31]. As depicted in Figure 5, no evidence of hemolysis was found in TACM group at different concentrations after 2 h of incubation, and the hemolysis rates of positive and negative groups were 100% and 0%, respectively. When the concentration reached 20 mg/mL, the hemolysis rate was still lower than 5%. Therefore, we concluded that the TACM could not cause hemolysis as an embolic material.

### 3.4. Biocompatibility of TACMs In Vivo

#### 3.4.1. Clinical and Biochemical Evaluation

**Figure 6:**
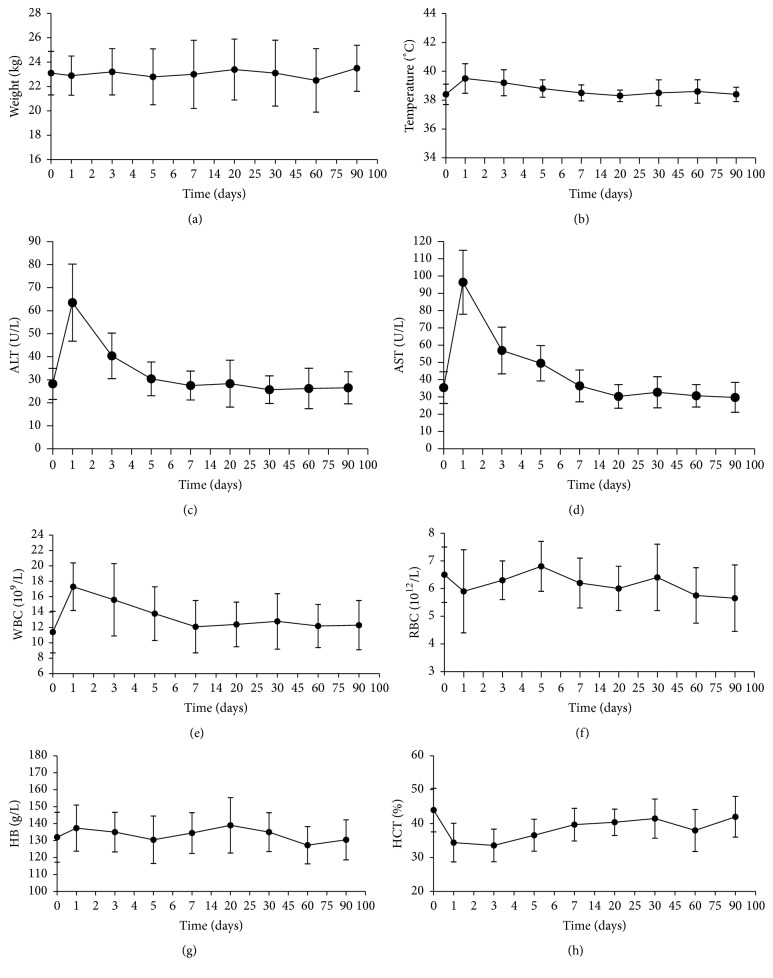
Clinical and biochemical changes of dogs after liver artery embolization. (a) Body weight values, (b) temperatures, (c) and (d) serum enzyme levels (ALT and AST), and (e)–(h) blood RT level (WBC, RBC, HB, and HCT) of dogs at 0, 1, 3, 5, 7, 20, 30, 60, and 90 d after operation.

Superselective embolization of live lobes was successful in all animals and they were in good health during the entire follow-up interval. Body weight change was an indicative parameter for toxic effects in laboratory animals [32, 33]. The mean body weight of animals in our research at different time points after embolization is given in Figure 6(a), and no obvious decline in body weight was observed. The changes in body temperature are shown in Figure 6(b), and no sign of fever (≥39.5°C) was found. According to the fluctuations of WBC in Figure 6(e), we can demonstrate that TACMs did not induce systemic inflammatory reactions. Although the serum ALT and AST levels were mildly elevated at 24 h, they declined gradually and were kept at a relative stable level 1 w after operation (Figures 6(c) and 6(d)). These results indicate that TACMs produce no toxic effects on liver tissue. In addition, the changes in RBC and HB levels after embolization were always kept within the normal range of variation (Figures 6(f)–6(h)).

#### 3.4.2. Histology Evaluation

**Figure 7:**
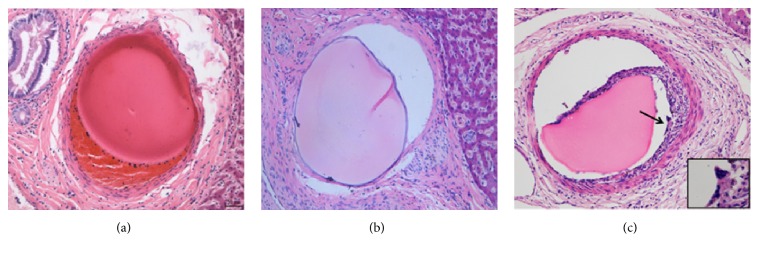
Tissue response of TACMs. (a) 48 h follow-up: Banff 97 score of 1; giant cell score of 0. (b) 4 w follow-up: Banff 97 score of 0; giant cell score of 0. (c) 12 w follow-up: Banff 97 score of 1; giant cell score of 1 (arrows and magnification indicate giant cells) (H&E, original magnification ×200).

Previous studies of materials composed of alginate mainly focused on implanting biomaterials into subcutaneous tissue or target organs by surgery and rarely involved tissue response around embolized artery [34–36]. In this study, we choose liver as an ideal organ for embolization because its dual blood supply would minimize necrotic or fibrotic tissue changes that might influence the onset of inflammatory cell and hinder following analysis [24, 37]. Banff 97 and giant cell scores were first raised by Stampfl et al. who used them to describe and compare material specific inflammation and foreign body reactions after porcine liver embolization [24]. Banff 97 score is well-standardized classification to identify vessel pathology associated inflammatory change, described as “arteritis,” and to assess the extent of inflammation around the embolic materials. In this paper, an overview of the nature and extent of tissue reactions as observed by light microscopic examination after implantation of TACMs is given in Figure 7 and Table 1, and there was no evidence of tissue necrosis and blood vessel injury at all time points (72 h, 4 w, and 12 w after operation). As shown in Figure 7(a), 72 h after embolization, a mild inflammatory reaction was observed around the embolized artery, which was mainly composed of neutrophils and lymphocytes, and there was some thrombus formation adjoined to the TACMs. 4 weeks after embolization (Table 1 and Figure 7(b)), Banff 97 score was likely to decline compared with 72 h group (Banff 97 score: 0.70 ± 0.46 versus 0.34 ± 0.47; *p* = 0.00), and a small number of infiltrating monocytes and lymphocytes responsible for chronic inflammation reaction were observed. Also it was observed that some macrophages were attached to the surface of microsphere, suggesting that TACMs were undergoing degradation. At 12 weeks (Table 1 and Figure 7(c)), the mean Banff 97 score was still low (0.28 ± 0.45), and no significant differences were found when compared with the 4-week group. Macrophages, giant cells, fibroblasts, and lymphocytes were found at the site of inflammation. Moreover, there were some granulation tissues around the TACMs, which were caused by thrombus organization.

After incorporation of foreign biomaterial, macrophages were attracted to implantation site and can fuse to form giant cells, releasing a multiplicity of substance and cytokines to degrade the foreign material and to activate additional inflammation [38, 39]. Anderson found that foreign body reaction can impact the biocompatibility of biomaterials and influence short- and long-term tissue responses [40]. Therefore, the extent of foreign body reactions should be observed. The giant cell score at overall time points is shown in Table 1 and Figure 7. Fewer giant cells were found at 72 h (giant score: 0.04 ± 0.19), and the number of giant cells slightly increased at 4 w and 12 w (giant scores: 0.30 ± 0.46 and 0.38 ± 0.49). The low giant cell scores found in our research show a good biocompatibility of TACMs in vivo. Several studies have investigated that the inflammatory response is closely intertwined with foreign body reaction, and giant cells modulate the inflammatory gene expression depending on the type of foreign material [41, 42]. Not surprisingly, there is a close correlation between the mild inflammatory reaction and the low giant cell score found in our study. In addition, we found a late appearance of giant cells and they kept infiltrating for several weeks or months, indicating that TACMs underwent slow degradation. The foreign body reaction is present for the in vivo lifetime of foreign material, and slow degradation can help maintain the occlusion and avoid hemorrhage after embolization.

### 3.5. The Results of Embolization Hemostasis In Vivo

The results of transcatheter application of TACMs mixed thrombus showed that the blood flow was shut down completely after the TACMs mixed thrombus was delivered to the injured hepatic artery without reflux of embolic agent. Most importantly, the postprocedural survival rate of all animals at 12 weeks was 100%. Angiography images were shown in Figure 1, the hemorrhage model of liver artery was established successfully (Figures 1(a)–1(c)), and continuous leakage of contrast agent from bleeding incision was shown. To achieve our goals of making embolization accurately and safely, the TACMs mixed thrombus was prepared in vitro and delivered by “sandwich delivery” method (Figure 1(d)). Figure 1(e) showed that the vascular channel distal to the embolism sites disappeared immediately and the leakage of contrast agent from the incision was not found any more after embolization. These results indicated that bleeding was successfully controlled by TACMs mixed clots. In addition, angiography in Figures 1(f)–1(h) showed that no definite recurrent hemorrhage of the injured hepatic arteries was found in any of animals during the 1 w, 4 w, and 12 w follow-up after embolization.

The current experiments with arterial embolization showed that TACMs have the advantage of no toxicity, ease of use, and long-term (12 weeks) reliable hemostasis. Thus, they have the potential to be a new ideal embolic material for interventional hemostasis in further clinical application. The hemostatic mechanism of TAMCs involves not only mechanical occlusion of mixed thrombin but also procoagulant activity of thrombin, sustainedly released from TACMs, which reinforce the mixed clots. In previous research, Rong et al. found that one-week survival rate after embolization in TACMs mixed clots group is significantly higher compared with autologous clots group [16]. The results of this research further indicated that the clot strength of thrombus could be increased by adding TACMs to form mixed clot.

Therapeutic benefits of TAE in hemorrhage are diverse depending on the amount of bleeding, risk of rebleeding, and the function of injured organ. Among them, the risk of rebleeding is a more important factor, especially in long-term recurrence. Thus, in our study, we set a follow-up period of 12 weeks to see if recurrent bleeding occurs. The results of this study illustrate a significant effect of stopping bleeding by using TACMs mixed thrombus at different time points. However, there are several inherent limitations in this study. First, the sites and severity of vascular injury seem to be inaccurate in our paper; thus, a standardized injury scale of blood vessel should be used in further study, especially when being compared with other embolic materials. Second, in this article, we merely performed a liver bleeding model to evaluate hemostatic efficacy of TACMs mixed thrombin. To further explore its clinical application, more complicated trauma models with different degrees of hemorrhage should be discussed.

## 4. Conclusions

In this study, we reveal good biocompatibility, effectiveness, and safety of the new embolic materials, TACMs. Combination of the excellent biocompatibility and effectiveness in vitro and in vivo experiments makes TACMs a promising candidate for embolic materials used in blunt abdominal trauma. However, further studies will be needed to compare the hemostatic efficiency of TACMs mixed clot with other currently available embolic materials.

## Figures and Tables

**Table 1:** Banff 97 inflammation score and giant cell score of TACMs.

	72 h follow-up (range)	4 w follow-up (range)	12 w follow-up (range)
Banff 97 score^a^	0.70 ± 0.46 (0-1)	0.34 ± 0.47 (0-1)	0.28 ± 0.45 (0-1)
Giant cell score^b^	0.08 ± 0.27 (0-1)	0.30 ± 0.46 (0-1)	0.38 ± 0.49 (0-1)

^a^Banff 97 score: 0 = no arteritis; 1 = mild-to-moderate intimal arteritis; 2 = severe intimal arteritis with at least 25% luminal area lost; 3 = transmural arteritis and/or arterial fibrinoid change and medial smooth muscle necrosis with lymphocytic infiltrate in vessel. ^b^Giant cell score: 0 = absence of giant cells; 1 = giant cells surround less than one-third of the particle circumference; 2 = giant cells surround >1/3 to <2/3 of the particle circumference; 3 = giant cells surround >2/3 of the particle circumference. The following comparisons were statistically significant: Banff 97 score: 72 h group versus 4 w group (*p* = 0.00); 72 h group versus 12 w group (*p* = 0.00); giant cell score: 72 h group versus 4 w group (*p* = 0.02); 72 h group versus 12 w group (*p* = 0.04). Data were presented as mean ± SD; ranges are in brackets [25].
